# Poland-Mobius Syndrome With Unilateral Vocal Cord Paralysis in a Neonate

**DOI:** 10.7759/cureus.10215

**Published:** 2020-09-02

**Authors:** Priyanka Yadav, Alpana Utture, Vaidehi Dande, Minnie Bodhanwala, Ankit Agarwal

**Affiliations:** 1 Neonatology, Bai Jerbai Wadia Hospital for Children, Mumbai, IND; 2 Head of institution, Bai Jerbai Wadia Hospital for Children, Mumbai, IND; 3 Pediatrics, Ascension Sacred Heart Hospital, University of Florida, Pensacola, USA

**Keywords:** mobius syndrome, poland syndrome, congenital

## Abstract

Poland-Mobius syndrome is a rare congenital disorder that includes features of Poland and Mobius syndromes. It is characterized by unilateral or bilateral congenital facial weakness, impairment of abduction of eyes, associated limb anomalies, and aplasia or hypoplasia of the pectoralis muscle. We describe a case of Poland-Mobius syndrome in a neonate associated with unilateral vocal cord immobility.

## Introduction

Mobius syndrome is a rare congenital disease characterized by unilateral or bilateral nonprogressive facial palsy (VII cranial nerve) with impairment of ocular abduction (VI cranial nerve). It can be associated with other cranial nerve palsies, limb defects, and orofacial anomalies [1]. The Poland syndrome presents with an absence of pectoralis major muscle, syndactyly, brachydactyly, and hypoplasia of the hand [2]. The combination of Mobius and Poland syndrome is rarely described in the literature with an estimated prevalence of 1:500,000. This report presents a rare case of a neonate with Poland-Mobius syndrome with unilateral vocal cord palsy.

## Case presentation

**Figure 1:**
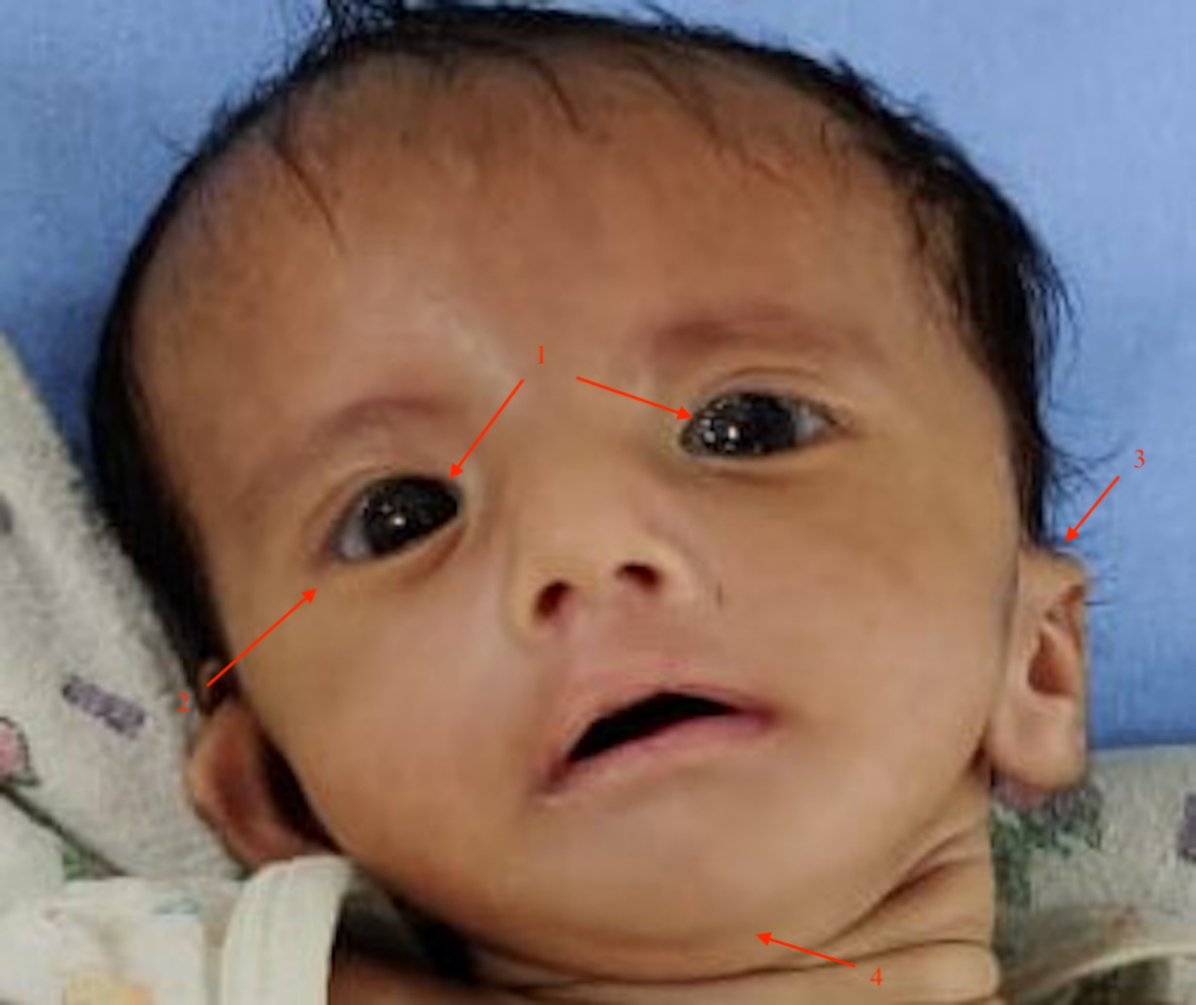
Image of the patient showing bilateral facial paralysis (lack of facial expressions) and other features of Poland-Mobius syndrome. 1: Bilateral abducens palsy; 2: Antimongoloid slant; 3: Low set ears; 4: Micrognathia

A 2,400-gm male infant was born at 37 weeks of gestation to a 44-year-old gravida 4 para 2 woman by vaginal delivery. There was no history of maternal drug intake, infection, or medical illness during pregnancy. Older siblings had no congenital disorder. The infant weighed 2,400 gm (>10th percentile), length 46 cm (>10th percentile), and head circumference was 34 cm (50th percentile). He was admitted to the hospital on day 3 of life with stridor and respiratory distress. On examination, the patient had suprasternal retractions and continuous positive airway pressure (CPAP) was initiated. Anomalies included bilateral facial paralysis (lack of facial expressions), antimongoloid slant, micrognathia, low set ears, and bilateral abducens palsy (Figure 1).

**Figure 2:**
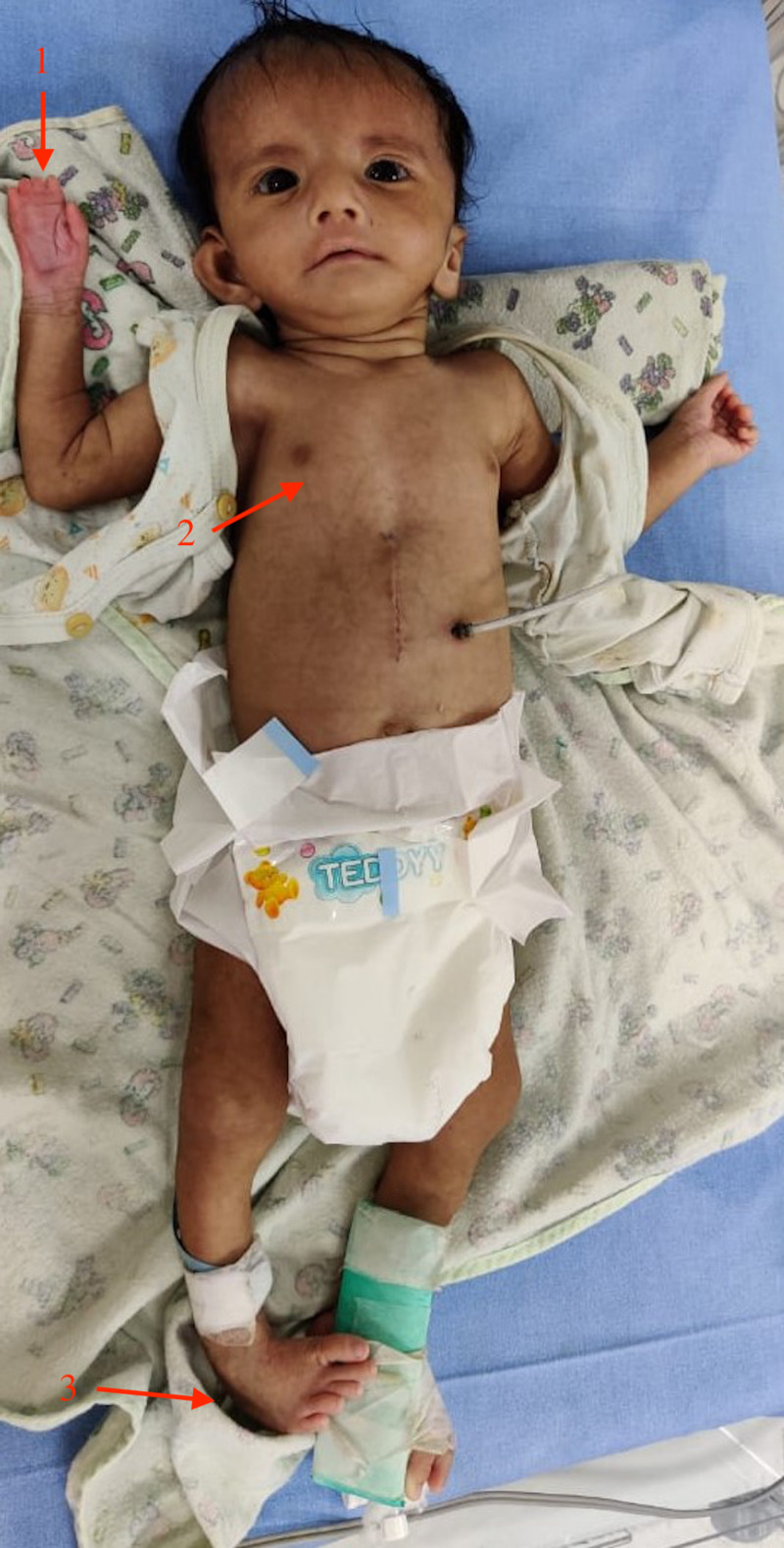
Image of the patient showing the features of Poland-Mobius syndrome. 1: Right hand syndactyly; 2: Hypoplasia of the right pectoralis major muscle; 3: Right foot talipes equinovarus

He had right hand syndactyly, hypoplasia of right pectoralis major muscle, and right foot talipes equinovarus (Figure 2).

**Figure 3:**
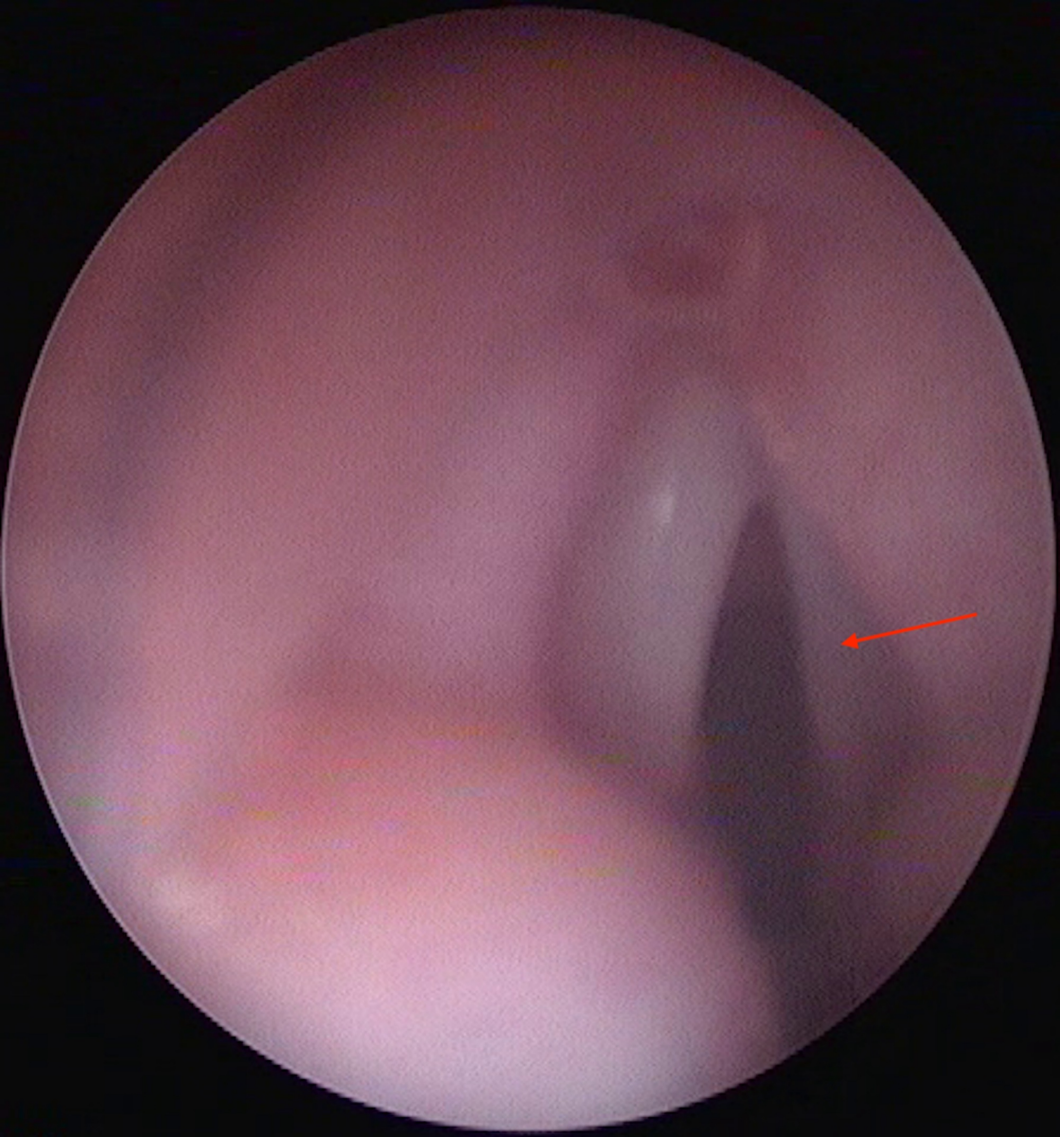
Direct laryngoscopy showing left vocal cord paralysis (red arrow).

Direct laryngoscopy showed left vocal cord immobility (Figure 3).

**Figure 4:**
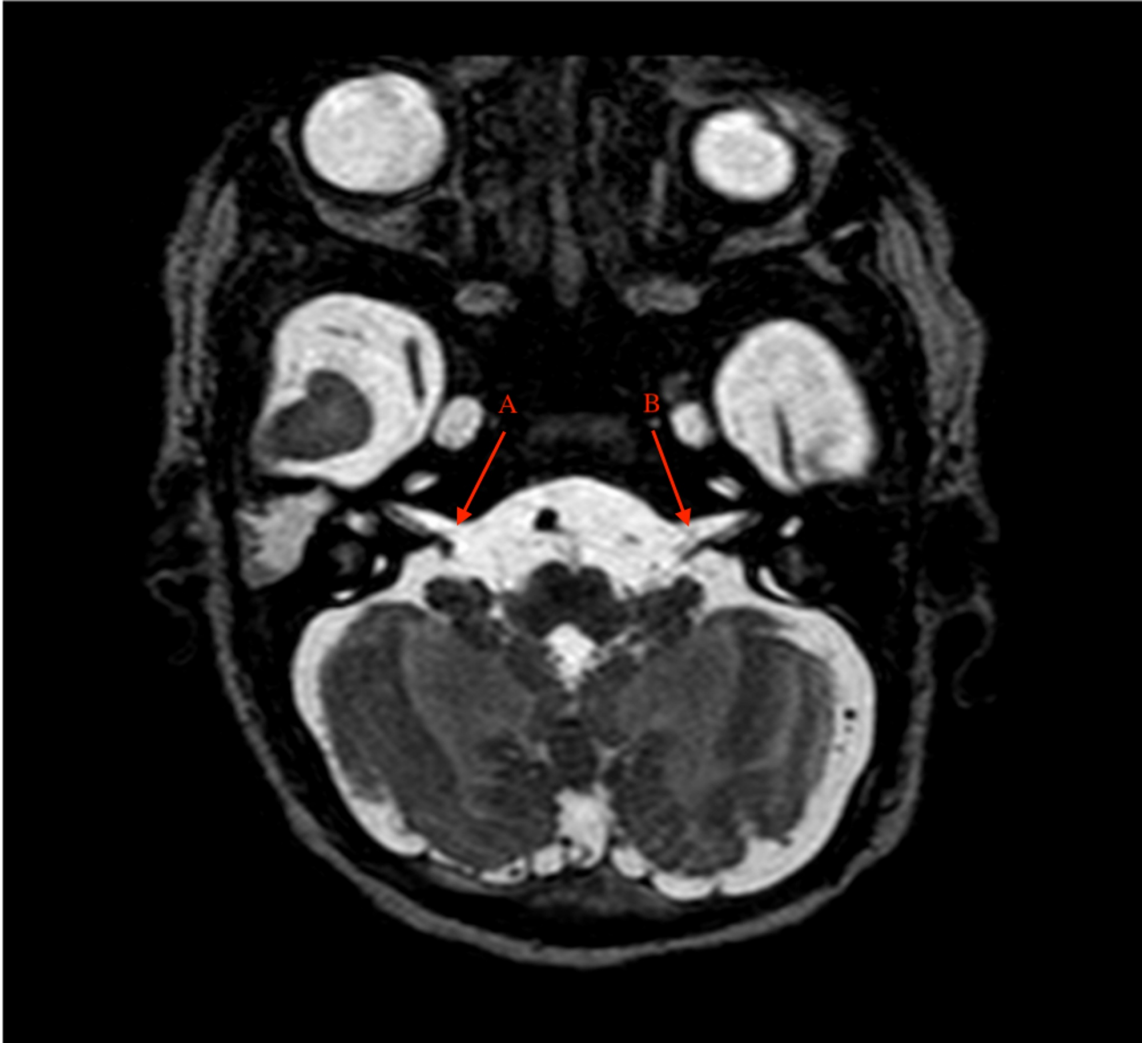
MRI brain showing absent right facial nerve (A) and hypoplastic left facial nerve (B).

Ultrasonography of chest showed absent right pectoralis major muscle. MRI of the brain showed hypoplasia of the sixth cranial nerve, absent right facial nerve, hypoplastic left facial nerve, and the absence of facial colliculi (Figure 4). 

Echocardiography and chromosomal analysis were normal. A feeding gastrostomy tube was placed as the patient had difficulty feeding due to absent gag reflex. As the respiratory status of the patient improved, the CPAP support was gradually weaned off in the next three days. A diagnosis of Poland-Mobius syndrome was suggested, and the patient was discharged.

## Discussion

Mobius syndrome is a rare congenital disorder that was first reported in 1880 by Von Graefe and by Paul Julius Mobius in 1888 [3]. It consists of unilateral or bilateral nonprogressive congenital facial palsy (VII cranial nerve) with or without paralysis of other cranial nerves. The most common cranial nerve involved is the abducent nerve (VI cranial nerve). Extraocular nerves, glossopharyngeal, vagus, and hypoglossal nerve may also be affected [4]. The suggested primary criterion for Mobius syndrome is facial palsy with impairment of ocular abduction. Dysfunction of other cranial nerves, orofacial malformations, limb malformations (syndactyly, brachydactyly, or absent digits and talipes), and musculoskeletal system defects are commonly associated features, but they are not necessary for the diagnosis [5]. Based on pathological changes, Mobius syndrome is classified into four groups [6]. Group I is characterized by simple hypoplasia or atrophy of cranial nerve nuclei, presumably as a result of embryonic maldevelopment. Group II results from primary lesions in the peripheral portion of the cranial nerves. Group III is due to focal necrosis in the brain stem nuclei. Group IV consists of patients without lesions in the central nervous system or cranial nerves but showed features of primary myopathy. The etiology and pathogenesis of Mobius syndrome remains undetermined; however, the triad of genetics, teratogens, and fetal vascular disruptions has been implicated [7]. An ischemic process at around four to six weeks of gestation resulting from an interruption in the vascular supply during early fetal development may result in facial and limb anomalies characteristic of Mobius syndrome [8]. Misoprostol, a synthetic prostaglandin E1, is an abortifacient agent that is used for legal and illegal abortions. There are case reports in the published literature showing unsuccessful attempts at abortion using misoprostol resulted in infants with Mobius sequence [9-11]. Cocaine, also known to cause uterine contractions, has been associated with a case of Mobius sequence [12]. Most cases of Mobius syndrome are sporadic but familial cases have been seen with both autosomal dominant and recessive patterns.

Poland syndrome was first described in 1841 by Alfred Poland as the unilateral absence of pectoralis major muscle and ipsilateral dermal syndactyly of the hand [13]. Other associated anomalies include hypoplasia of the forearm, hypoplasia of the breast, agenesis of the nipple, rib cage deformities, atrial septal defect, bilateral epicanthus, telecanthus, and talipes equinovarus [2]. The incidence is 1 in 30,000 births and boys are affected more than girls. The syndactyly is usually in the right hand as seen in our patient. The etiology and pathophysiology of Poland syndrome is unclear, although a vascular injury to the subclavian artery has been hypothesized [14]. The condition is seldom familial, but the transmission mechanisms are still unknown [15].

Treatment for Mobius syndrome is supportive and in accordance with symptoms. Infants may require a feeding tube to facilitate meeting nutritional needs. Surgery may improve limb and jaw deformities. Treatment for facial paralysis may require a variety of surgical procedures, such as fat or muscle transfers, fascia slings, lid weights, and Botox®. The goals of therapy remain to restore facial symmetry, protect the eye and blinking capability, provide oral continence, and allow for the expression of emotion [7]. Physical and speech therapy often improves motor skills and coordination with better control of speaking and eating abilities. Likewise, surgical reconstruction is indicated for patients with Poland syndrome with significant deformities of the chest wall and the overlying soft tissue [16].

Unilateral or bilateral vocal cord palsy is a significant cause of upper airway obstruction leading to stridor, respiratory distress with suprasternal retractions in a neonate. It may be caused by birth trauma, asphyxia, or central nervous system disorders. Neonates with suspected vocal cord paralysis must be evaluated for underlying diseases. Flexible laryngoscopy is used for the diagnosis of vocal cord immobility. To our knowledge, no previous cases of Poland-Mobius syndrome with congenital unilateral vocal fold paralysis have been reported in the literature.

## Conclusions

Although the pathogenesis of Mobius syndrome is unclear, toxic, genetic, intrauterine vascular, and infectious factors have been proposed as causative. Evidence for the vascular disruption theory is that different causes of premature uterine contraction early in pregnancy have been associated with Mobius infants. In our case, neither family history nor teratogen exposure was found. This case was classified as a sporadic Poland-Mobius syndrome with unilateral vocal cord paralysis.
